# Fall Detection in Elderly People: A Systematic Review of Ambient Assisted Living and Smart Home-Related Technology Performance

**DOI:** 10.3390/s25216540

**Published:** 2025-10-23

**Authors:** Philippe Gorce, Julien Jacquier-Bret

**Affiliations:** 1 University of Toulon, CS60584, Cedex 9, 83041 Toulon, France; gorce@univ-tln.fr; 2 International Institute of Biomechanics and Occupational Ergonomics, Avenue du Docteur Marcel Armanet, CS 10121, 83418 Hyères Cedex, France

**Keywords:** fall detection, elderly, AAL, smart home, wearable and non-wearable sensors, accuracy, precision, sensitivity, specificity, F1-score

## Abstract

**Highlights:**

**What are the main findings?**

- Non-wearable sensors and hybrid solutions (wearable + non-wearable sensors) achieved the highest fall detection performance.
- Deep learning methods produced the best performance results.

**What are the implications of the main findings?**

- Propose a systematic review of fall detection systems’ performances.
- Identify the advantages of different solutions in terms of performance for researchers, practitioners, and policymakers in order to design and implement more effective fall detection systems.

**Abstract:**

Fall detection systems in ambient assisted living (AAL) and smart homes are essential for the comfort, safety, and autonomy of elderly people. The aim of this study was to investigate the performance of these systems considering categories of sensors and methods used. A systematic review was conducted following the Preferred Reporting Items for Systematic Reviews and Meta-Analyses (PRISMA) guidelines. Seven open databases were screened without a date limit: PubMed/MedLine, Google Scholar, ScienceDirect, Science.gov, Academia, IEEE Xplore, and Mendeley. The article selection and data extraction were performed by two authors independently. Among the 473 unique records, 80 studies were selected. Five fall detection performance parameters (accuracy, precision, sensitivity, specificity, F1-score) and two computation speed parameters (training and testing time) were extracted and classified according to three sensor categories (wearable, non-wearable, and hybrid solutions), and four methods (deep learning, machine learning, threshold, and all others). The ANOVA results showed that wearable sensors performed the worst in fall detection. Deep learning methods produced the best results for the five parameters. Identifying the advantages of different solutions is a major challenge for researchers, practitioners, and policymakers in the design and implementation of more effective fall detection systems.

## 1. Introduction

Improvements in quality of life have led to increased life expectancy and, as a result, a rise in the proportion of elderly people worldwide. Currently, there are over 1 billion people aged over 60. According to the United Nations, this number is expected to double by 2050 (2.1 billion) and triple (3.1 billion) by 2100 [1]. Preserving their autonomy and quality of life is a major challenge.

Falls are a major cause of injuries that can have dramatic consequences. They directly impact comfort and independence and generate significant costs for healthcare systems. For example, in 2014, in the USA, 29 million falls among older Americans caused seven million injuries and medical expenses of $31 billion [2]. In the European Union, more than €25 billion is spent on fall-related healthcare costs each year, an expenditure that is set to increase to approximately €45 billion by 2050 due to aging populations across the continent [3]. In China, the direct medical cost of falls among older adults is more than 5 billion yuan per year [4]. Consequently, extensive research and development on fall detection systems are essential to ensure safety, particularly at home.

Ambient Assisted Living (AAL) is a concept that uses technology to improve the quality of life and well-being of elderly people. Its aim is to preserve their independence and autonomy while ensuring their safety. Activity recognition is the most widely used methodology in the field of assisted living technology. It involves automatically detecting and classifying activities performed by occupants, including falls, using sensors. Researchers have used various methodologies based on different sensor types. The most common are wearable sensors. Their small size allows them to be integrated into clothing, watches, or other devices. They collect physical and contextual information for local processing or direct transmission to the central unit of an AAL system [5]. These include accelerometers, gyroscopes, magnetometers, and orientation sensors. They are often integrated into inertial measurement units [6,7] or portable devices such as bracelets [8,9] or smartwatches [10,11]. They are also found in smartphones, which are used alone or in combination with biological and behavioral monitoring [12,13] due to their high number of integrated sensors and cost-effectiveness [14,15]. Other non-wearable sensors have been used to detect falls without being attached to the body. These solutions play a crucial role in the functionality and effectiveness of AAL systems. Operating passively, these sensors autonomously monitor the occupants of a room without manual intervention. They include all camera systems (RGB [16], Kinect [17], etc.), radio frequency sensors [18,19], radars [20], vibration sensors [21], thermal sensors [22], and infrared sensors [23]. Other authors have combined wearable and non-wearable sensors to offer hybrid solutions [24,25].

Regardless of the sensors used, the data was processed using algorithms of different complexity. The simplest fall detection techniques are based on fixed [26] or adaptive [27] threshold detection. Thresholds are also used in hybrid versions to trigger a sensor (e.g., a video camera) when they are reached [25]. Over the last decade, Machine Learning (ML) methods have used more advanced algorithms that exploit larger databases to train different classifiers to identify a fall. Support Vector Machines (SVMs) [28,29], Artificial Neural Networks (ANNs) [30,31], K-Nearest-Neighbors (KNNs) [32,33], and Decision Trees (DTs) [34,35] are among the most widely used. More recently, Deep Learning (DL) methods have emerged. They automatically identify the characteristics necessary for detecting or classifying an activity from raw data, without significant human intervention [36]. DL methods mainly use four categories of algorithms, i.e., Convolutional Neural Networks (CNNs) [37,38], Long Short-Term Memory (LSTM) [39,40], Recurrent Neural Networks (RNNs) [41,42], and auto-encoders [43].

Fall detection technologies must be reliable and effective to ensure an appropriate response. The authors evaluated the performance of their algorithms using quantitative parameters such as accuracy, precision, specificity, sensitivity, and the F1-score. These parameters are computed from the confusion matrix, which contains the number of true and false positives and negatives [38,44]. Some studies have used one [45,46] or several [47,48] of these parameters. They have also evaluated processing speed performance using training times and testing times [49].

Several studies have proposed a state-of-the-art overview of existing techniques and algorithms in this field. Two recent studies conducted bibliometric analyses. Li et al. [50] proposed a synthesis dedicated to fall detection using wearable sensors over the last 10 years. Sanchez et al. [51] investigated work focused on Human Activity Recognition (HAR) in the AAL and smart home fields based on 100 most cited studies. Other works have been presented as reviews focusing on a specific point of view of fall detection. Casilari-Pérez et al. [52] and Iadarola et al. [53] provided a detailed summary of techniques based on wearable sensors, either solely in the AAL context [53] or by reporting performance information in a more general context [52]. Other authors have focused on methods. Amir et al. [54] summarized studies using ML (without specifically focusing on AAL and elderly people), while Islam et al. [55] and Gaya-Morey et al. [56] studied DL methods. Only the study by Islam et al. [55] reported performance parameters but not in an AAL context. Guerra et al. [57] provided a state-of-the-art overview of the different types of HAR sensors in AAL, reporting the method used but without focusing on performance. Finally, Ren et al. [58] produced a similar analysis, adding performance information but without being in the AAL context.

To our knowledge, no study in the literature has investigated the performance of fall detection systems in AAL. This requires consideration of different types of sensors (i.e., wearable sensors, non-wearable sensors, and hybrid solutions) and detection methods (i.e., threshold, ML, and DL). The objective of this study was to examine whether there was a relationship between the type of sensor or method used and performance parameters (detection, learning speed, and detection speed). The major contributions would be to present the quantitative values of the different performance parameters grouped by sensor category, method, and evaluation datasets in order to identify the most effective. Given the importance of AAL systems for the autonomy of elderly people, identifying the advantages of different solutions is a major challenge for researchers, practitioners, and policymakers in the design and implementation of more effective fall detection systems.

## 2. Related Works

In the context of fall detection, AAL system monitoring solutions can be based on a variety of technologies. There are three main categories of sensors: wearable sensors, non-wearable sensors, and hybrid solutions that include both of the previous categories [57]. Wearable devices generally consist of small sensors that can be integrated into clothing, accessories such as jewelry, and more recently into electronic devices such as watches and smartphones. They collect information that is processed locally or transmitted directly via appropriate communication protocols to the central unit of an AAL system [5]. Accelerometers and gyroscopes are the most commonly used portable sensors and are often combined in an inertial measurement unit [59]. They measure linear acceleration and angular velocity in their own local three-dimensional coordinate system. They are an attractive solution because they are inexpensive, compact, and can continuously measure motion-related parameters in real time [60] with high precision and accuracy [61]. These sensors were used to study the effect of different sensor positions [38] when detecting falls or optimizing energy expenditure [62]. Two approaches are commonly used to detect falls: threshold-based and artificial intelligence-based, which mainly use machine learning and deep learning methods. Pham et al. [63] used the fixed threshold approach based on accelerometer data to detect falls recorded in young people. They achieved an accuracy of 92%, a sensitivity of 93.3%, and a specificity of 91.4%. Based on similar data, Wu et al. [64] used an adaptive threshold approach with a sensitivity of 90% and a specificity of 92%. Yu et al. [65] used an ML method and obtained very high-performance parameters, based on accelerometers (sensitivity of 100% and specificity of 99.8%). Santos et al. [38] used CNN-type DL algorithms that produced performance between 99% and 100% for accuracy, specificity, sensitivity, and precision on the URDF dataset.

Non-wearable sensors play a fundamental role in the effectiveness of AAL systems. They include devices that enable passive recognition of human activities, i.e., without being directly worn by individuals. They are deployed in a room and autonomously monitor the occupants within it. Unlike wearable systems, these solutions require a more extensive infrastructure, including power and data cabling, wired or wireless connection to a data storage and processing server, and a setup and calibration phase prior to use, which often entails higher costs [66]. Vision-based fall detection, mainly different types of cameras (RGB, depth, infrared, etc.), hold an important place among non-wearable sensors. Li et al. [67] used Kinects combined with CNN-type DL analysis and achieved detection performance close to 100% (99.98% accuracy, 100% sensitivity, and 99.98% specificity). Shu et al. [68] used a machine learning-derived relevance vector machine algorithm to detect falls using cameras with a training accuracy of 94%. Other studies have used environmental sensors such as thermal, infrared, acoustic, or ultrasonic sensors. Bharathiraja et al. [69] deployed a system using AMG8833 infrared thermal sensor detection. In this technique, the sensor provided a set of temperature data corresponding to thermal signatures that are affected by changes in environmental conditions. Popescu et al. [70] used KNN (ML) approach on data recorded by three acoustic sensors to identify the fall detection. They had a sound level information system to achieve 100% fall detection. Kittiyanpunya et al. [71] used a millimeter-wave frequency-modulated continuous wave radar. They transformed the radar scattering signals into point clouds and Doppler velocity data. With an LSTM network, they achieved 99.5% accuracy in fall detection. Several studies have also explored floor-based fall detection. Clemente et al. [72] used seismic sensors placed on the floor of an apartment. Using SVM analysis, the authors detected falls with 73% accuracy and 86% sensitivity.

Finally, hybrid solutions coupling the two categories of portable and non-portable sensors have been implemented. They combine the advantages of each solution while limiting the disadvantages, which in principle increases the ability to detect falls. The most commonly used combination associates inertial data with video analysis. Kwolek et al. [25] have used information from a Kinect and an inertial measurement unit to detect falls. Detection is carried out in two stages: the inertial measurement unit detects a potential fall based on movement (threshold analysis), and the fall is then confirmed using an SVM classifier applied to the Kinect’s depth images. Using their methods, the authors achieved 98.33%, 96.77%, 100%, and 96.67% for accuracy, precision, sensitivity, and specificity, respectively. Li et al. [73] proposed a similar approach to fall detection based on accelerometer data recorded by a smartphone and video data captured by a Kinect. The authors indicated that their fall detection approach achieves the best accuracy of 100%. Although the high level of performance is achieved in part by the redundancy and complementarity of the information, hybrid solutions nevertheless have certain limitations. The use of different types of sensors requires more sophisticated algorithms, which increase the complexity of processing (synchronization, multiple layers, etc.) and require greater storage capacity. The installation and configuration of these solutions are also more complex (cables, synchronization, communication with the central server, etc.). All these factors have an impact, particularly on cost, which becomes more expensive.

## 3. Materials and Methods

The protocol was registered in the PROSPERO (CRD420251136411) and PRISMA (Preferred Reporting Items for Systematic reviews and Meta-Analyses) guidelines proposed by Harris et al. [74] and Moher et al. [75] was followed to report the present systematic review results.

### 3.1. Search Strategy

The aim of this research was to investigate the performance of fall detection systems for elderly people in an AAL context. All generations of AAL systems, i.e., wearable sensors, non-wearable sensors, and hybrid solutions, were included in the analysis. The performance of the different systems was evaluated using the following parameters: accuracy, sensitivity, precision, F1-score, or training and testing time. To achieve this objective, a detailed list of keywords linked by the logical operators AND and OR, parentheses, and the operator “*” was used in seven open databases: PubMed/MedLine, Google Scholar, ScienceDirect, Science.gov, Academia, IEEE Xplore, and Mendeley. Due to differences in the search engines of each database, the list of keywords had to be slightly adapted to each one. Table 1 details the search strategy for each database explored. The research was conducted between June 22 and July 10, 2025, and all entries were considered without any date restrictions.

Data management was conducted as follows: (1) all items found in each database were merged into a single table (Microsoft Excel file); (2) the automatic detection function was used to remove multiple entries; (3) two independent reviewers (PG and JJB) separately evaluated the title and abstract of each unique entry to identify relevant works according to the inclusion/exclusion criteria; (4) the results were compared to establish the list of articles to be evaluated based on their full text; (5) the two reviewers evaluated the remaining full-text articles separately. Any article did not meet the inclusion/exclusion criteria were excluded; (6) the list of remaining articles from each reviewer was compared to determine the final list of articles to be included. All discrepancies were resolved by rereading the article and discussing to reach a consensus.

The second stage of the selection process used the snowball method. During the full-text evaluation, the list of references of each study was analyzed. A list of potentially relevant works was compiled. Each study was then evaluated, and those that met the selection criteria were manually added to the list of included works.

### 3.2. Selection Criteria

Several inclusion criteria were applied to select studies that met the objective. First, the studies had to be conducted in the context of AAL or smart homes for the elderly. Only published peer-reviewed original studies were considered. The reported method had to enable fall detection and had to include the following information: sensors, methods, and datasets used. The performance data had to be available throughout quantitative parameters: accuracy, precision, specificity, sensitivity, F1-score, training and testing time.

The exclusion criteria were: (1) the study design was a conference, book or chapter, review, report, case report, case study; (2) language of publication was not English; (3) the article has not been peer-reviewed; (4) the sample was not old people; (5) insufficient method detail or no performance parameters available.

### 3.3. Data Extraction and Classification

The data collected for each included article were as follows: first author name, year of publication, type of sensors used (accelerometer, camera, radar, acoustic sensor, etc.), their positions (on the body or external), their technical characteristics (range, sample, etc.), the method, algorithms and datasets used, type of input data, detection performance parameters (accuracy, precision, sensitivity, specificity, F1-score) and computation speed (training and testing time) when available.

To make it easier to read a large amount of information, the data was reported in summary form using abbreviations and classified. First, the data was presented by sensor category, i.e., wearable, non-wearable, and hybrid solutions, as proposed by Guerra et al. [57]. The different methods were presented according to the three families traditionally used in the literature: DL, ML, and Threshold [54,57]. Any study that did not correspond to any of these families was classified under the heading “Other”. Regarding algorithms, we considered all solutions in each method (CNN, SVM, KNN, LSTM, DT, etc.). For fall detection performance, the five most commonly used parameters in the literature were considered: accuracy (Acc), precision (Prec), sensitivity (Sens), specificity (Spec), and F1-score (F1) [47,48]. They are obtained from the four information items contained in the confusion matrix: true positives (TP, correctly predicted positives), true negatives (TN, correctly predicted negatives), false positives (FP, incorrectly predicted positives), false negatives (FN, incorrectly predicted negatives). The formulas were: Accuracy = (TP + TN)/(TP + TN + FP + FN), Sensitivity = TP/(TP + FN), Specificity = TN/(FP + TN), Precision = TP/(TP + FP), F1-score = 2 × (Sensitivity × Precision)/(Sensitivity + Precision). For computing speed, training and testing time values were used [49,76].

### 3.4. Data Analysis

In order to meet the objective of assessing the link between performance and fall detection systems, we summarized the results as follows. First, the five performance parameters were presented by sensor category, i.e., wearable, non-wearable, and hybrid solutions. Second, these same five parameters were displayed by method, i.e., DL, ML, Threshold, and Other. For each method, the performance parameters were plotted according to the most commonly used algorithms across all included studies. A third analysis reported the results of the five performance parameters according to the datasets used to test the methods proposed in the articles. Finally, training and testing times were analyzed by method and then detailed according to the most commonly used algorithms. In each section, the data were averaged across all included studies for which data were available.

### 3.5. Statistical Analysis

The effect of sensor categories (wearable, not wearable, and hybrid solution) and methods (ML, DL, Threshold, and Other) on fall detection performance parameters (Acc, Spec, Sens, Prec, and F1) was investigated using statistical analyses. First, the normality of the data distribution was checked using the Shapiro–Wilk test and the homogeneity of variances using the Levene test. In case these two conditions were verified, a one-way analysis of variance (ANOVA) was applied and Tukey’s post hoc test was used to identify the differences. The effect size was assessed using Cohen’s d. Otherwise, a non-parametric analysis was conducted using the Kruskal–Wallis test. Differences were analyzed using Dunn’s post hoc test with Bonferroni’s adjusted *p*-value and effect size was estimated with ε^2^ parameter. The significance level of all tests was set at 5%. All analyses were performed using JASP software (JASP Team, v0.19.3, Amsterdam, The Netherlands).

## 4. Results

### 4.1. Search Results

The search identified 484 items in the seven databases, with 11 duplicates. From the 473 unique items found, 280 were excluded because studies were conference, book, thesis, report, review, survey, or bibliometric analysis or did not deal with fall detection. Among the remaining 193 articles, 18 reports were not retrieved and 144 were excluded based on the selection criteria, i.e., no static and dynamic analysis, no performance values (accuracy, specificity, sensitivity, or precision values), or no training or testing time. Thus, the selection procedure retained 31 articles.

**Figure 1:**
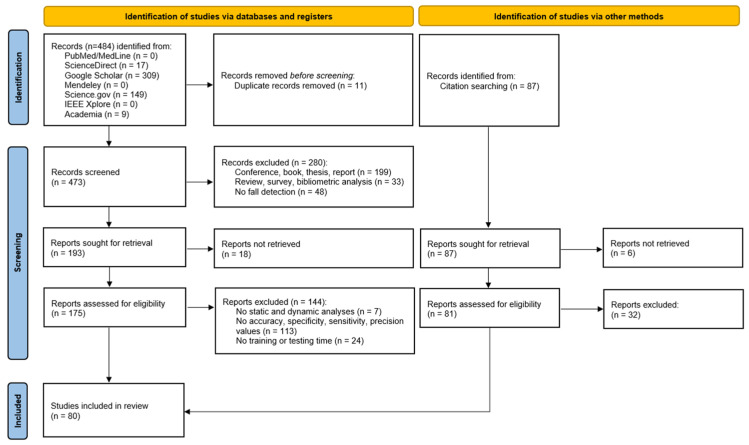
PRISMA flow diagram.

A second search was conducted based on citation searching (snowballing). 87 articles were identified and 38 were excluded (6 reports not retrieved and 32 excluded based on the selection criteria). Thus, 49 articles were added to the 31 identified by the search. A total of 80 articles were therefore included in the present study. Figure 1 depicts the results of the selection process in detail.

### 4.2. Study Characteristics

Table 2 presents information on the sensors, methods, and algorithms used in each study selected. In the 43 studies using wearable sensors, nine different sensors were reported: accelerometer (the most commonly used), angular velocity sensor, electrocardiogram, electromyogram, gyroscope, magnetometer, magnetic sensor, orientation sensor, and pressure sensor. All parts of the body were used to position the various sensors, with the majority located on the wrist or waist. For non-wearable sensors, seven different devices were identified in the 30 studies: antennas and receivers, acoustic sensors, different types of cameras (the most commonly used), infrared sensors, radar, thermal sensors, and vibration sensors. For the seven studies that used a hybrid approach, a combination of accelerometers, gyroscopes, magnetometers, and cameras was chosen. In terms of methodology, ML and DL were mainly implemented in the studies, with a wide variety of algorithms. CNN and LSTM were the most commonly used in DL, while SVM and KNN were the most commonly used in ML. Two trends emerged in the evaluation of the methods used: either the authors tested one or more open datasets (SisFall, MobiFall, PAMAP2, WISDM, USC-HAD, etc.), or they experimentally developed their own dataset (denoted “custom” in the table). Thirty-three different datasets were found in the included studies. The type of input data is labeled “Dynamic” in the case of a time series and “Static” in other cases [57]. In summary, the most used wearable sensors were accelerometers (86% of studies) and gyroscopes (34.9% of studies). For non-wearable sensors, the camera was used much more than all other sensors (53.3%; radar: 16.7; acoustic sensor: 13.3%). For the hybrid solution, the camera + accelerometer combination was used in all the studies included. For the methods, DL was the most used for non-wearable sensors (47% of the included studies) while ML was the most used for wearable sensors (44.2% of the studies). However, for these two categories, there are many other methods (Others: 47% and 55.8% of the studies, respectively). The threshold method was very few used (only 14% for wearable sensors and 28.6% for hybrid solutions). The few studies with a hybrid solution used all the methods. Regarding the datasets, a large part of the studies used their own data (60.47% for wearable sensors and 70% for non-wearable sensors). For the others, the most used datasets were SisFall, MobiFall, PA-MAP2, WISDM, USC-HAD.

Table 3 shows the performance parameters reported by each study, classified by sensor type. The presence of the X symbol indicates that the authors evaluated their method or algorithms using the considered parameter. In many studies, the authors tested several methods or algorithms on one or more datasets. Thus, several values for the same parameter were reported in a single study. The data used in the present study to evaluate performance was detailed in the Supplementary File. It contains 426 accuracy values across 59 studies, 363 sensitivity values from 50 studies, 351 specificity values from 52 studies, 229 precision values across 25 studies, 232 F1-score values from 19 studies, 50 training time values, and 28 testing time values from 9 and 8 studies, respectively. Thus, accuracy (wearable: 67.4%; non-wearable: 80.0%; hybrid: 100% of included studies) and specificity (wearable: 74.4%; non-wearable: 60.0%; hybrid: 28.6% of included studies) were the two most frequently evaluated parameters and the F1-score the least measured. Training and testing time parameters were very rarely reported (less than 15% of studies).

### 4.3. AAL Fall Detection Performance per Sensor Category

**Figure 2:**
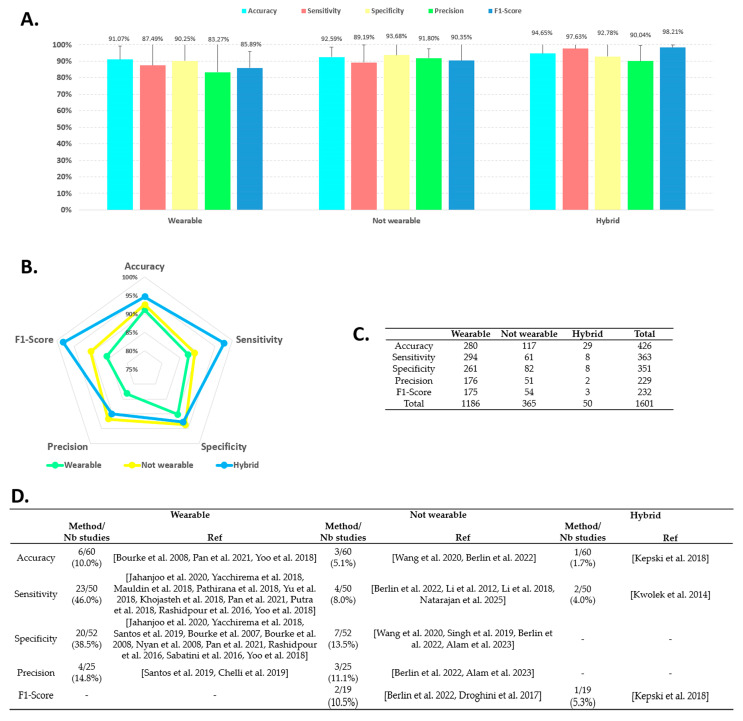
Fall detection performance analysis based on sensor category. (**A**): Histogram of performance parameter distribution by sensor category. (**B**): Radar chart comparing sensor categories for the five performance parameters. (**C**): Table listing the number of data entries available across all studies by parameter and sensor category. (**D**): Table displaying the distribution of studies that achieved 100% performance by parameter and sensor category. Nb = number; Ref = references. References: [15,19,22,35,38,41,48,61,65,79,80,83,88,94,96,97,98,101,107,110,112,120,121,128,134].

Figure 2 presents the analysis of performance parameters by sensor category, i.e., wearable sensors, non-wearable sensors, and hybrid solutions. Parts A and B detail the values of the performance criteria, Table C indicates the number of available values, and Table D shows the number of studies that achieved 100% for each performance parameter. The effect of sensor category was observed for 4 of the 5 parameters. The accuracy of the hybrid solution was higher compared to wearable sensors (94.6% vs. 91.1%, F = 6.314, *p* < 0.05, ε^2^ = 0.015). Non-wearable sensors scored an accuracy of 92.6%. Hybrid solution also showed the best sensitivity performance (97.6%, F = 8.049, *p* < 0.05, ε^2^ = 0.022) statistically higher than wearable (87.5%) and not wearable sensors (89.2%). For precision (F = 12.324, *p* < 0.05, ε^2^ = 0.054), and F1-score (F = 17.572, *p* < 0.05, ε^2^ = 0.076), the non-wearable sensor category achieved significantly higher performance than the wearable sensor category (91.8% vs. 83.3%, 90.3 vs. 85.9%, respectively). For these two parameters, the values measured for the hybrid solution were 90.0%, and 98.2% (statistically higher than wearable, *p* < 0.05), respectively. Only specificity had no effect (F = 3.537, *p* > 0.05; hybrid solution: 92.8%; non-wearable sensors: 93.7%; wearable sensors: 90.2%). Table D illustrates the number of studies that achieved 100% performance: 9 for accuracy, 29 for sensitivity, 27 for specificity, 7 for precision, and 3 for F1-score. This number was higher for the wearable sensor category when considering accuracy, sensitivity, specificity, and precision. Sensitivity and specificity provided the best results, with rates of 46.0% (23/50) and 38.5% (20/52) of studies, respectively.

### 4.4. AAL Fall Detection Performance per Method

**Figure 3:**
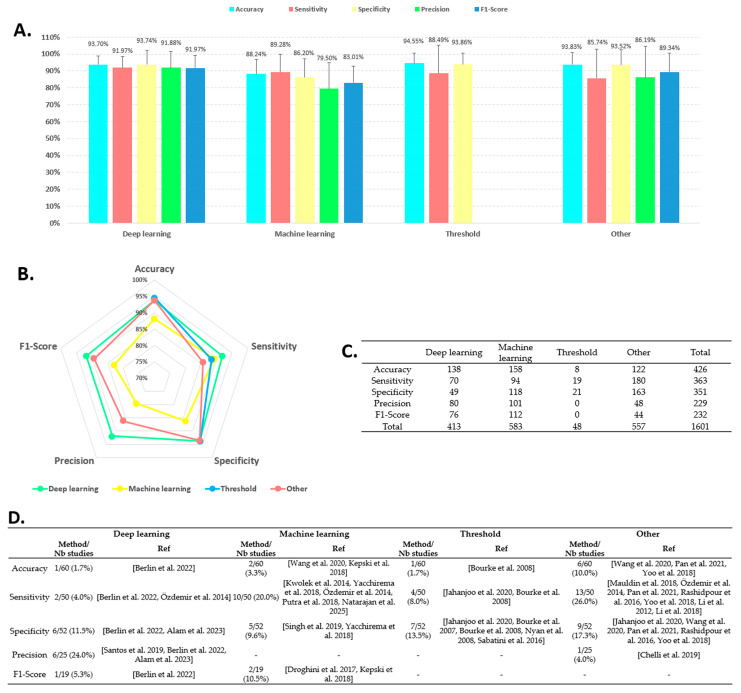
Fall detection performance analysis based on methods. (**A**): Histogram of performance parameter distribution by methods. (**B**): Radar chart comparing the four methods for the five performance parameters. (**C**): Table listing the number of data entries available across all studies by parameter and method. (**D**): Table displaying the distribution of studies that achieved 100% performance by parameter and method. Nb = number; Ref = references. References: [15,19,22,25,35,38,41,48,76,79,80,83,94,96,97,98,101,107,110,112,120,121,128,134].

Figure 3 presents the results of fall detection performance system for DL, ML, Threshold, and other methods separately. As in Figure 2, parts A and B depict the values of the performance criteria, Table C and D detail the number of available values and the number of studies that achieved 100% performance for each method, respectively. An effect of methods was observed on all performance parameters. The accuracy of the DL (93.7%), Threshold (94.5%), and Other (93.8%) methods showed similar results. The three methods performed significantly better than ML (88.2%, F = 45.897, *p* < 0.05, ε^2^ = 0.108). The same results were observed for specificity: DL (93.7%), Threshold (93.9%), and Other (93.5%) showed similar results that were statistically higher than the ML (86.2%, F = 45.491, *p* < 0.05, ε^2^ = 0.130). No effect was found for sensitivity. No data were available for the Threshold in terms of precision and F1-score. For these two parameters, the DL (91.9% and 91.8%) and Other methods (86.0% and 89.3%) performed significantly better than the ML (79.5% and 83.0%, F = 61.740 and F = 41.730, respectively, *p* < 0.05, ε^2^ > 0.2). In summary, DL produced the best results for all five parameters. Table C shows the distribution of the available values by method in the 80 studies included. ML was the most represented method with 583 values, followed by the Other category (557 values) and DL (413 values). Table D details the distribution of studies that achieved 100% performance: 10 for accuracy, 39 for sensitivity, 27 for specificity, 7 for precision, and 3 for F1-score. All parameters combined, the DL, ML, and Threshold methods presented an equivalent number of algorithms that achieved 100% performance (between 12 and 19 studies). Sensitivity was the parameter that presented the best results with 26% (13/50) and 20% (10/50) studies for Other and ML, respectively.

### 4.5. AAL Fall Detection with 100% Performance

Table 4 presents all studies that reported at least one performance parameter at 100% by sensor category: fifteen studies for wearable sensors, eight for non-wearable sensors, and two for hybrid solutions. The two performance parameters for which the largest number of studies reported a value of 100% were sensitivity and specificity. Berlin et al. [48] were the only ones to propose a detection method with all five parameters at 100%. The authors used a camera (non-wearable sensors) and a 2D-CNN to detect falls. Three studies [81,96,107] achieved 100% accuracy, sensitivity, and specificity using wearable sensors (mainly an accelerometer).

### 4.6. AAL Fall Detection Performance per Algorithm

**Figure 4:**
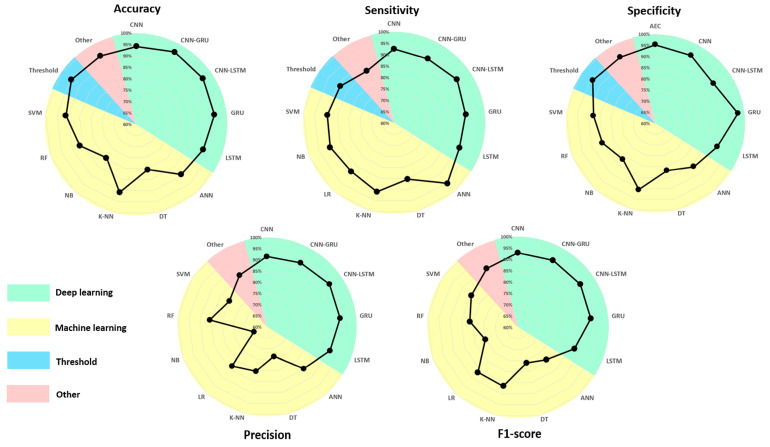
Radar chart of most commonly used algorithm performance for DL, ML, Threshold, and other methods.

Figure 4 illustrates the performance of the most commonly used algorithms in each method. For DL, CNN, LSTM, Gait Recurrent Unit (GRU) algorithms and their combination (CNN-GRU and CNN-LSTM) showed performance ranging from 90 to 95% for the five performance parameters. The GRU algorithm achieved a specificity of 97.13% whereas LSTM produced an F1-score of 86.62%. For ML, the most commonly used algorithms were SVM, RF, NB, KNN, DT, ANN, and LR, with mixed results. For accuracy, SVM, KNN, and ANN produced the best results (≥90%), while NB and DT reported values around 80%. For sensitivity, ANN performed best (95.33%) and DT reported the lowest results (85.35%). The other algorithms scored around 90%. For specificity, KNN showed the best results (90.58%), while the others achieved scores close to 85%. The least effective were NB and DT, with 81% specificity. Three algorithms showed greater precision values: ANN (84.84%), RF (85.60%), and LR (83.32%). The others had results ranging from 80.34% (SVM) to 66.17% (NB). Finally, for the F1-score, KNN (86.62%) and LR (86.36%) had the best scores, followed by SVM (84.79%). For the others, the F1-score was around 75%.

### 4.7. AAL Fall Detection Performance per Datasets

**Figure 5:**
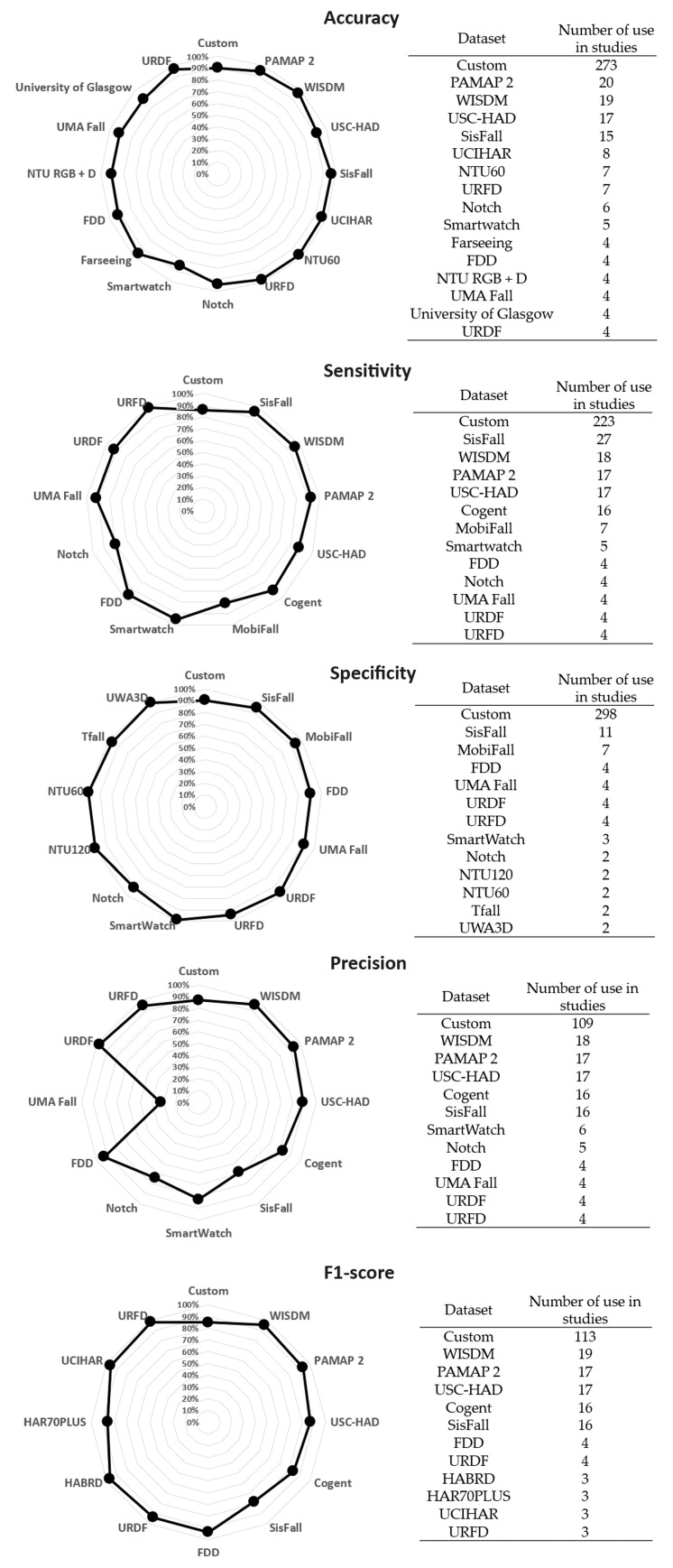
Radar chart of fall detection performance computed by dataset for the five parameters.

The performance of the algorithms was evaluated in two different ways. The authors either developed their own datasets based on experimentation (“custom” Table 2) or used an existing database dedicated to fall detection. The most commonly used were WISDM (Acc = 19 values; Sens = 18 values; Prec = 18 values; F1-score = 19 values), PAMAP 2 (Acc = 20 values; Sens = 17 values; Prec = 17; F1-score = 17 values), USC-HAD (Acc = 17 values; Sens = 17 values; Prec = 17; F1-score = 17 values), Cogent (Acc = 0; Sens = 16 values; Prec = 16 values; 1-score = 16 values), and SisFall (Acc = 15 values; Sens = 27 values; Spec = 11 values; Prec = 16 values; F1-score = 16 values). Figure 5 displays the performance results for the datasets used at least 4 times.

Two-thirds (273/426) of the accuracy values were obtained with a “Custom” dataset for an average of 90.33%. The others (153/426) were obtained by evaluating the methods using 29 different datasets. For the most frequently used, the accuracy ranged from 91.5% (USC-HAD) to 97.2% (WISDM). The best performance was obtained with the NTU120 dataset at 99.84%, but it was only used twice.

Sixty percent (223/363) of sensitivity values were found with a “Custom” dataset for an average of 85.84%. The others (140/363) were computed from 19 different datasets. For the most commonly used, sensitivity ranged from 87.5% (USC-HAD) to 95% (WISDM). The best sensitivity was reached with the URFD dataset at 99.10%, used four times.

Eighty-five percent (298/351) of the specificity values were achieved with a “Custom” dataset, with an average of 90.44%. The others (53/351) were obtained by evaluating the methods with 18 different datasets. Among the most commonly used, only SisFall had a value of 94.72% (11 datasets). The best specificity was found with the GMDCSA dataset, with 100% used only once.

Half (109/229) of the precision values were obtained with a “Custom” dataset, with an average of 86.98%. The remaining values (120/229) were computed from 18 different datasets. For the most commonly used, the precision ranged from 68.5% (SisFall) to 96% (WISDM). The best performance was found with GMDCSA dataset, with 100% precision, used only once.

Half (113/232) of the F1-score values were found with a “Custom” dataset, with an average of 84.88%. The others (119/232) were obtained by evaluating the methods using 22 different datasets. For the most commonly used, the F1-score ranged from 78% (SisFall) to 95.5% (WISDM). The best F1-score was obtained with the URFD dataset with 98.21% used three times.

### 4.8. Algorithm Training and Testing Time

**Figure 6:**
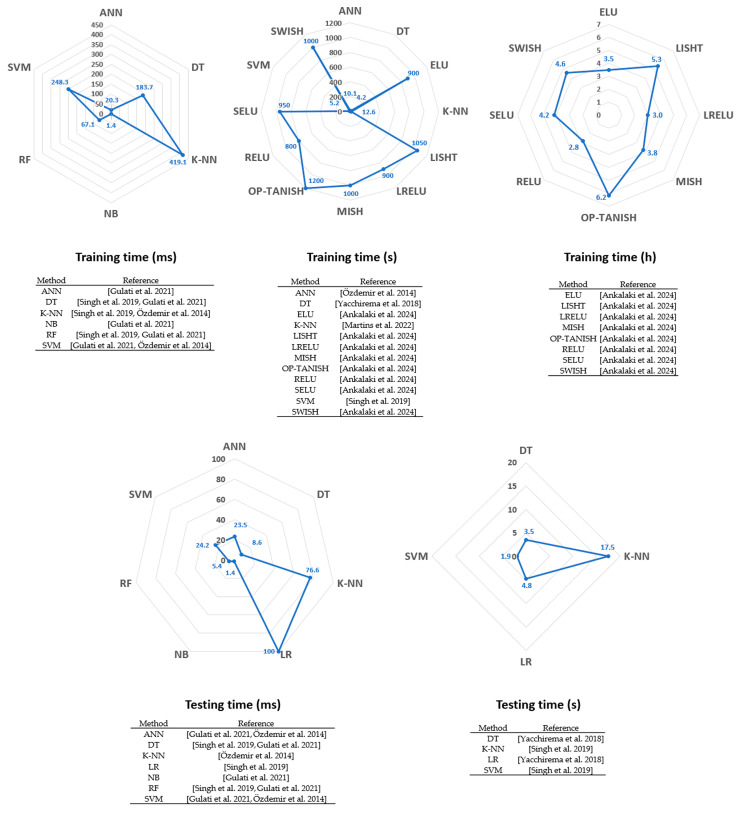
Radar chart of training and testing time by algorithm. The studies were divided into units of equal length. References: [22,35,49,76,78,91].

The data showed strong heterogeneity. Three studies reported times in ms [22,49,76], five studies in s [22,35,76,78,91], and one study in hours [78]. Training times ranged from 1.4 ms (NB algorithm [49]) to 6.2 h (OP-Tanish algorithm [78]) from 9 studies whereas testing times ranged from 1.4 ms (NB algorithm [49]) to 17 s (KNN [22]) from 8 studies. Figure 6 separately presents the training and testing times in milliseconds, seconds, and hours available in the 80 included studies for the different fall detection algorithms. 

## 5. Discussion

The objective of this systematic review was to provide an overview of the fall detection systems for elderly people in an AAL and smart home context and to evaluate their performance. Knowledge of this performance is essential in order to choose the solution best suited to the environment and user constraints. The analysis was conducted by considering wearable, non-wearable, and hybrid technologies, as well as different methods based on ML, DL, and Threshold for fall detection, based on 80 selected studies.

### 5.1. Performance by Sensor Category

Currently, studies using wearable sensors have been the most numerous (43/80). This is consistent with the bibliometric results proposed by Li et al. [50], which showed that the number of studies using this technology has increased steadily over the last decade. The most common sensors were accelerometers, gyroscopes, and magnetometers. They are found in inertial measurement units and in technologies that are increasingly present in everyday life, such as smartwatches [41,136] and smartphones [137,138]. This is due to technological advances that have made possible the miniaturization of equipment and improvements in the performance of this sensor category, offering a discreet monitoring solution. However, the effectiveness of these wearable devices depends heavily on their use. For older adults, this is not necessarily the most suitable solution, as they often forget to use additional accessories or resist using them [139]. In addition, the issue of energy dependence is a major drawback. These constraints can be compensated for by the use of non-wearable sensors, which were found in 30 of the studies included in this analysis. The most common were cameras, which can be of various types [126,127,140,141]. Radars, infrared, pressure, vibration, and acoustic sensors were also used (Table 2). They present the advantage of being placed in the environment and therefore do not encumber individuals. However, they are fixed devices that can only cover the environment for which they have been calibrated. In addition, these devices are sensitive to occlusions, which can lead to a loss of information and make setup difficult when multiple sensors are present [57]. In AAL or smart homes, the increase in the number of sensors provides a good understanding of the environment but leads to an increase in the cost of the fall detection system. Finally, more recently, hybrid solutions combining wearable and non-wearable sensors (mainly accelerometers and cameras) have appeared, and there were seven of these in our study. The fusion of sensors offers a robust approach, as data from different types of sensors can complement each other, producing a reliable system [142]. However, these solutions have several drawbacks. On a technical and technological level, the fusion of data from heterogeneous sensors requires sophisticated algorithms to integrate and synchronize the different formats and poses the problem of data storage [57]. Indeed, the multiplication of sensors and video is resource-intensive. In addition, the presence of several types of devices requires more significant maintenance (management of the batteries of wearable sensors, calibration of fixed sensors, cleaning, replacement or recalibration of damaged or obsolete equipment, etc.). Finally, to operate at full capacity, it is necessary that all sensors operate simultaneously. Forgetting to wear or the failure of a wearable sensor or the obstruction of a fixed sensor can lead to an increased risk of false alarms or an undetected fall. From an economic point of view, hybrid solutions require the purchase (or periodic replacement) and installation of several sensors associated with a reliable network (cabling, wireless communication system, configuration, etc.) which represents a higher cost than a wearable or non-wearable solutions.

In the present study, the results showed that the overall performance of the three categories of sensors was around 90%. Hybrid systems displayed all five performance parameters above this value (90.04% to 98.21%). Non-wearable sensors achieved a sensitivity of 89.19%, while the other parameters were between 90.35% and 93.68%. Finally, wearable sensors showed three parameters below 90%: sensitivity (87.49%), precision (83.27%), and F1-score (85.89%). This level of performance appears satisfactory, with an advantage for hybrid solutions. However, these results should be adjusted for several reasons. Statistical analysis revealed that hybrid solutions had only significantly higher accuracy than wearable systems, but with an overall score above 90% for all three categories. For specificity, precision, and F1-score, the non-wearable sensor category achieved higher performance than the wearable sensors. Finally, no effect was found for sensitivity despite a 10% higher performance for hybrid solutions. On the other hand, solutions with 100% performance for one or more parameters were found in each of the three categories.

### 5.2. Performance by Methods

Fall detection uses the three methods commonly used in the literature, i.e., threshold, ML, and DL [57,58]. The advantage of threshold-based algorithms is their low computational complexity. In the case of wearable sensors, they are generally associated with data acquisition and preprocessing [143], while for non-wearable sensors, they are directly integrated into the central host that receives and processes the data [14]. The main disadvantage lies in the definition of the threshold, as it is sensitive to inter- and intra-individual variability as well as to the sensor location. Incorrect threshold definition could lead to a decrease in the accuracy of fall detection [144]. The other two methods use intelligent approaches to detect falls. Supervised or unsupervised algorithms are applied to large databases to train a classification method. At the end of this learning stage, the classifiers become able to identify a fall [145]. The main difference is that ML requires a large amount of manually extracted information, whereas DL automatically extracts relevant features [41,146]. The need for large databases for learning, dependence on data quality (performance declines in the case of noisy or incomplete data), often significant hardware requirements (memory, processors, etc.), and the loss of ability to generalize on new data in the case of over-specialized learning are the main disadvantages of these methods [54,57].

In this study, performance results were compared. An overall performance of 90% was observed for all methods. More specifically, the Threshold and Other methods achieved results above 90% in terms of precision and specificity, while for DL, all five parameters were above this value. ML presented the lowest values, with all five parameters below 90%. This trend needs to be adjusted. Statistical analysis showed that the DL, Threshold, and Other methods had significantly higher precision and specificity than the ML method. For sensitivity, the DL methods performed significantly better than the other methods. Precision and F1 score of the DL (91.9% and 91.8%) and Other (86.0% and 89.3%) methods performed significantly better than the ML method. In summary, ML methods perform the least well, while the other methods appear to be equivalent, with DL performing slightly better. This result is consistent with several studies that have shown that DL methods performed better [40,147]. However, solutions with 100% performance have been identified in the literature for the different methods (Figure 3). In particular, across all performance parameters, ML methods account for the highest number of algorithms with 100% performance.

### 5.3. Algorithms

For ML, the most commonly used algorithms were ANN, KNN, DT, NB, and SVM. For DL, the most commonly used were CNN, LSTM, GRU, and their combinations. On average, DL algorithms produced the best results. This has been observed in several studies comparing different algorithms from ML and DL methods [87,148].

### 5.4. Datasets

A total of 63.5% of the methods and algorithms identified in this analysis were evaluated on the basis of a custom dataset, i.e., obtained experimentally. The others were assessed using 33 open datasets available in the literature (WISDM, PAMAP 2, USC-HAD, and SisFall for the most commonly). The various results reported showed very high average performance, >90% for the large majority of studies across all parameters. This can be explained by the fact that many studies use part of the dataset to train the algorithms and the other part to perform tests [149,150]. As the data was collected under similar conditions, identification could be facilitated, thereby providing effective fall detection. The use of different datasets for training and testing algorithms could be a first step in testing algorithms in situations occurring under different conditions. It should be noted that the available datasets or custom datasets use fall simulations, which may partly explain false negatives or false positives.

### 5.5. Training and Testing Time

Ten studies reported in this analysis measured the training time of the algorithms (for ML and DL) and the fall detection time (testing time). The results showed a wide range of durations, from 1.4 ms to several hours. For training, a long duration may not be problematic as long as the detection is fast. On the other hand, a method with an excessively long detection time could have significant consequences for the safety of elderly people in their environment and make these methods unsuitable for real-time situations.

### 5.6. Limitations

The first limitation concerns the conditions for evaluating the performance of fall detection systems. Indeed, this systematic review has shown that the datasets used to compute the various performance parameters were highly heterogeneous, whether they were available in the literature or constructed experimentally. This variability in the datasets has a major impact on the performance, regardless of the system itself (sensors and methods/algorithms used). This point is reinforced for ML and DL methods, as they are often trained with data extracted from the evaluation datasets. Standard evaluation conditions are necessary to accurately compare different systems. Finally, even though the datasets used have improved, they still offer only stereotypical solutions to falls, often simulated, without considering the health profile of participants or changing environments.

The second limitation concerns the performance assessment by category of wearable, non-wearable, and hybrid sensors. The results highlighted their respective advantages. However, the analysis could be extended by divided sensors type in subgroups: accelerometers, gyroscopes, cameras, etc. The same recommendation can be made for fall detection methods (mainly ML and DL), for which subgroups by algorithm (SVM, KNN, LSTM, RF, CNN, etc.) could be compared.

Another limitation concerns the analysis of training and testing times for fall detection systems. Few studies have reported this performance data, and often the test conditions (a single computational module vs. a complete method) in each study were very different or only partially described.

The final limitations are methodological and relate to the criteria for selecting articles. On the one hand, the research focused on fall detection systems for elderly people in the AAL context. In addition, the research was conducted using selected keywords, for which not all synonyms were necessarily used. On the other hand, only original peer-reviewed research written in English was included. These choices may have led to the omission of some relevant studies in which the fall detection system was effective.

### 5.7. General Outcomes and Future Research Directions

As we have seen in this study, fall detection systems are evolving with technological advances and often offer viable solutions with overall performance (across all performance parameters studied) of around 90%. Some solutions even achieve 100% for all parameters. Despite this, these detection systems remain imperfect and require further research and development. Future advances could focus on predicting falls before they occur [151] by exploiting behavioral and movement anomalies in older people. Another approach is not to simply study falling from a standing posture but to generalize it to complex behaviors such as activities in a sitting position or during physical activities (jogging, cycling, etc.). Systems should therefore have more sophisticated predictive algorithms to help anticipate a fall and take preventive measures. Furthermore, with regard to wearable systems and hybrid solutions, acceptability should be considered by offering solutions designed to guarantee long-term comfort, ergonomics, and ease of use. This is achieved through the design of intuitive interfaces and lightweight, miniaturized solutions [152,153]. Improving energy autonomy of wearable sensors is also essential to ensure continuous real-time monitoring without frequent recharging. For non-wearable sensors and hybrid solutions, privacy concerns will need to be addressed. There is a need for continued technical development and research to address ethical and social acceptability issues. The Internet of Things (IoT) is a promising avenue for fall detection [154]. The development of smartwatches, smartphone apps, and, more recently, connected clothing are all solutions that make fall detection more discreet and, a priori, more acceptable. The integration of sensors into furniture also contributes to this invisible detection. Connections to various healthcare services allow fall situations to be communicated in real time and contribute to the safety and protection of the elderly. However, the cost of these new systems will need to be controlled and managed, as it could be a barrier in regions where access to healthcare technologies is limited. All these improvements will make the use of these fall detection systems more attractive and acceptable to elderly people.

It could be considered to establish standards for system reliability and security. The first could consist of identifying a list of universal performance parameters that would allow the proposed solutions to be compared within a common framework. Commonly used parameters such as accuracy, precision, specificity, sensitivity, and F1-score are a good starting point but need to be supplemented by others. Similarly, the situations used as a basis for evaluation should be standardized and rely on real fall data rather than simulations, as is the case in many available datasets. This would enable objective comparison of different methods and testing their respective performance. Fall detection time is also a very relevant and important parameter, but it needs to be obtained within a well-defined context. This indicator is rarely tested in real-life situations. It contributes to the safety and protection of people in AAL environment. Finally, data security is also crucial for the viability of these systems. The development of secure data storage and transmission methods is essential for protecting users’ privacy.

All these advances could lead to the development of high-performance, adaptive systems that could consider the user’s health profile, level of independence and activity, and environment.

## 6. Conclusions

This literature review contributes to the field by providing an overview of the performance of fall detection systems. Its originality lies in its presentation by sensor category and detection method. Identifying the advantages of different solutions provides a source of information for researchers, practitioners, and policymakers in the design and implementation of more effective fall detection systems that are accepted by users. By combining these performance-related aspects with the challenges of cost, ergonomics, energy management, and privacy, future solutions could help improve the independence, safety, protection, and care of older adults.

## Figures and Tables

**Table 1:** Keyword combination for each database.

Database	Keyword Combination
PubMed/Medline	“wearable sensors”, (“Monitoring” OR “Ambient Assisted Living” OR “Assist* Living” OR “AAL” OR “Smart Home”), (“fall detection” OR “fall prevention” OR “fall risk assessment”), (“Static” OR “dynamic”), (“Elder*” OR “Senior” OR “old* people”), (“training time” OR “testing time”), (“specificity” OR “accuracy” OR “sensitivity” OR “precision”)
Google Scholar	“wearable sensors”, (“Monitoring” OR “Ambient Assisted Living” OR “Assist* Living” OR “AAL” OR “Smart Home”), (“fall detection” OR “fall prevention” OR “fall risk assessment”), (“Static” OR “dynamic”), (“Elder*” OR “Senior” OR “old* people”), (“training time” OR “testing time”), (“specificity” OR “accuracy” OR “sensitivity” OR “precision”)
ScienceDirect	“wearable sensors” AND “Ambient Assisted Living” AND “fall detection” AND “training time” AND “Elderly” AND accuracy AND specificity
Science.gov	Wearable sensors, fall detection, AAL, elder, accuracy
Academia	“wearable sensors”, “Ambient Assisted Living”, “fall detection”, accuracy, sensitivity, specificity
IEEE Xplore	sensor elderly AAL fall
Mendeley	wearable sensors AND Ambient Assisted Living AND fall detection AND training time AND Elderly AND accuracy AND sensitivity AND specificity

**Table 2:** Detailed presentation of sensors, methods, algorithms, and datasets for each of the 80 studies included. Data are classified by sensor category: wearable sensors, non-wearable sensors, and hybrid solutions.

Authors	Sensor Type	Sensor Position	Sensor Characteristics	Used Method	Algorithms	Used Dataset	Input Data Type
**Wearable sensors**							
Agrawal et al., 2023 [77]	P	Foot	20 Hz	ML	SVM, RF, LR, NB, DT, KNN	Custom	
Al-Hassani et al., 2023 [43]	A, G, O		100 Hz	DL	AEC	Custom	
Ankalaki et al., 2024 [78]	A, G, M	Various		DL	CNN	UCIHAR, PAMAP2, Opportunity, Daphnet Gait HAR, UPFALL, SIMADL	
Bourke et al., 2007 [79]	A		±10 g	Threshold	Threshold	Custom	Dyn
Bourke et al., 2008 [80]	G	Chest	G: 1 kHz	Threshold	Threshold	Custom	
Butt et al., 2021 [81]	ECG	Chest		DL	CNN	Custom	
Chandramouli et al., 2024 [82]				DL	CNN, LSTM	Actitracker, MHEALTH	
Chelli et al., 2019 [83]	A, G	Chest	A: ±8 g, 100 Hz, G: ±2000°/s, 100 Hz	ML, Other	KNN, SVM, ANN	Custom	
Chen et al., 2018 [84]	A		±2 g to ±4 g, 96.35 to 202.1 Hz	Threshold	Threshold	Custom	Stat
Gibson et al., 2016 [47]	A	Chest	50 Hz	ML, Other	ANN, KNN	Custom	
Gulati et al., 2021 [49]	A, G	Wrist		ML	RF, SVM, NB, DT, ANN	ADL, ARFall	Dyn
He et al., 2016 [34]	A, G	Neck	A: ±16 g, G: ±2000°/s	ML, Other	KNN, NB, ANN, DT	Custom	
He et al., 2017 [85]	A, G		A: ±16 g, 100 Hz, G: 2000°/s, 100 Hz	ML, Other	KNN, NB, DT	Custom	
Jahanjoo et al., 2020 [15]	A	Waist		Threshold	Threshold	tfall, MobiFall	
Jantaraprim et al., 2012 [86]	A	Chest	1 kHz	Other		Custom	
Kerdegari et al., 2015 [87]	A	Waist	±3 g, 100 Hz	Other		Custom	
Khojasteh et al., 2018 [88]	A	Wrist, Waist	16 to 204.8 Hz	ML, Other	DT, SVM	UMAFall	
Kraft et al., 2020 [89]	A	Wrist, Waist		DL	CNN	Notch, MUMA, SimFall, Smartwatch, Smar-Fall, UPFall	
Liaqat et al., 2021 [90]	A	In the pocket		ML, DL, Other	LR, RF, KNN, SVM, DT, MLP, CNN, LSTM	Custom (experimental, 30 subjects, 6 ADL)	Stat
Martins et al., 2022 [91]	A, G, M	Lower back, Thighs, Waist, Foot		ML, Other	KNN	Sisfall, FallAIID, FARSEEING, UCI HAR, UMAFall, Custom	
Mauldin et al., 2018 [41]	A	Wrist, Waist	±8 g to ±16 g, 21.25 to 100 Hz	ML, Other	NB, SVM	Smartwatch, Notch, Farseeing	
Medrano et al., 2017 [92]	A		50 Hz	ML, Other	SVM	tfall	Stat
Miah et al., 2024 [93]	A, G, M			ML, DL, Other	SVM, HMM, GRU, CNN, LSTM	WISDM, PAMAP2, USCHAD, Opportunity, UCI HAR	
Nyan et al., 2008 [94]	A, G	Waist, Thigh	A: ±4 g, G: 150°/s	Threshold	Threshold	Custom	
Özdemir et al., 2014 [76]	A, G, M	Head, Chest, Waist, Wrist, Thigh, Ankle	A: ±13 g, 25 Hz, G: ±1200°/s, 25 Hz, M: ±1.5 Gauss, 25 Hz	ML, Other	KNN, SVM, ANN	Custom	
Özdemir et al., 2016 [95]	A, G, M	Head, Chest, Waist, Wrist, Thigh, Ankle	A: ±13 g, 25 Hz, G: ±1200°/s, 25 Hz, M: ±1.5 Gauss, 25 Hz	ML, Other	KNN, SVM, ANN	Custom	
Pan et al., 2021 [96]	A, AV, Mag	Shoulder, Waist, Foot		Other		Custom	
Putra et al., 2018 [97]	A	Chest, Waist	100, 200 Hz	DL	CNN	Cogent, Sisfall	
Rashidpour et al., 2016 [98]	A, G	Thigh	A: ±2 g, 87 Hz, G: ±2000°/s, 200 Hz	Other		MobiFall	
Ren et al., 2016 [99]	A	Waist	62.5 Hz	Other		Custom	
Rescio et al., 2018 [100]	EMG	Leg	1 kHz	Other		Custom	
Sabatini et al., 2016 [101]	A, G, M	Waist	A: ±4 g, 50 Hz, G: 2000°/s	Threshold	Threshold	Custom	
Santos et al., 2019 [38]	A	Wrist, Waist	±8 g to ±16, 21, 25 to 256 Hz	DL	CNN, LSTM	URFD, Notch, Smartwatch	
Sarabia-Jácome et al., 2020 [102]	A	Waist	±16 g, 100 Hz	ML, DL, Other	LSTM, GRU, SVM, KNN	SisFall	Dyn
Shahzad et al., 2018 [103]	A	Waist, Thigh	64 Hz	ML	SVM, ANN, KNN, NB	Custom	
Suriani et al., 2018 [104]	A	Hip, Thigh, Foot	±3 g, 50 Hz	ML	KNN, SVM	Custom	
Torti et al., 2019 [42]	A			DL	LSTM	SisFall	Dyn
Tunca et al., 2019 [105]	A, G, M	Foot		ML, DL	SVM, RF, MLP, HMM, LTSM	Custom	
Wu et al., 2018 [64]	A, AV		A: ±16 g, 20 Hz, G: 2000°/s, 100 Hz	Other		Custom	
Xi et al., 2017 [106]	EMG	Thigh, Leg	1024 Hz	Other		Custom	
Yacchirema et al., 2018 [35]	A	Waist		ML, Other	DT, SVM, MLP, KNN	SisFall	
Yoo et al., 2018 [107]	A	Wrist	50 Hz	Other		Custom	Dyn
Yuwono et al., 2012 [108]	A	Right pocket	±6 g, 20 Hz	Other		Custom	
**Not wearable sensors**							
Adnan et al., 2018 [109]	AS	Ext	16 to 48 kHz	ML	SVM	Custom	
Alam et al., 2023 [110]	C	Ext		DL	CNN	CAUCAFall, GMDCSA	
Berlin et al., 2022 [48]	C	Ext		DL	CNN	URFD, FDD	
de Miguel et al., 2017 [111]	C	Ext		ML	KNN	Custom	
Ding et al., 2023 [20]	R	Ext		DL, ML, Other	CNN, KNN, LSTM	Custom	
Droghini et al., 2017 [46]	AS	Ext	44,100 kHz	ML	SVM	Custom	
Droghini et al., 2017 [112]	AS	Ext	44,100 kHz	ML	SVM	Custom	
Fan et al., 2017 [113]	C	Ext		ML, Other	MLP, SVM	Custom	
Guerra et al., 2020 [114]	C	Ext		Other		Fall detection, Fall detection testing	Stat and Dyn
Guerra et al., 2022 [115]	C	Ext		DL	GRU, LSTM	Custom	Dyn
Guerra et al., 2023 [17]	C	Ext		DL	LSTM	Custom	Dyn
Helen Victoria et al., 2021 [116]	R	Ext	400 MHz, 5.8 GHz	DL	CNN	University of Glasgow	
Hu et al., 2014 [117]	C	Ext	100 Hz	Other		Custom	
Huu et al., 2022 [118]	C	Ext		DL, ML, Other	SVM, CNN, LSTM	Human pose, Custom	
Karayaneva et al., 2023 [119]	C	Ext		DL, Other	CNN, LSTM	Custom	Stat and Dyn
Li et al., 2012 [120]	AS	Ext	20 kHz	Other		Custom	
Li et al., 2018 [121]	C	Ext		Other		Custom	
Li et al., 2020 [122]	R	Ext	7.3 to 25 GHz	DL	LSTM	Custom	
Li et al., 2021 [123]	C	Ext		Other		NTU RGB + D	Dyn
Li et al., 2023 [124]	R	Ext		DL, Other	CNN, LSTM	Custom	
Liu et al., 2019 [28]	V	Ext		Other		Custom	
Martelli et al., 2014 [125]	C	Ext	100 Hz	DL	CNN	Custom	Dyn
Min et al., 2018 [126]	C	Ext	256 Hz	DL	CNN	URFD, Custom	
Min et al., 2018 [127]	C	Ext		ML	SVM	TST Fall	
Natarajan et al., 2025 [128]		Ext		ML	SVM	Custom	
Qiao et al., 2022 [129]	R	Ext		DL, Other	CNN	Custom	
Singh et al., 2019 [22]	T	Ext		ML	LR, SVM, KNN, DT, RF	Custom	Stat
Spasova et al., 2016 [130]	IR	Ext		ML	SVM	Custom	Stat
Wang et al., 2020 [19]	A + R	Ext		ML, Other	HML	Custom	
Zahan et al., 2021 [131]	C	Ext		DL, Other	CNN	UWA3D, NTU60, NTU120	
**Hybrid solution**							
Alabdulkree et al., 2023 [132]				DL, Other	CNN	Custom	
Benhaili et al., 2025 [45]	A, G, C	Waist	A: 50 Hz, G: 50 Hz, C: 200 Hz	DL, Other	CNN, LSTM, GRU	ICU HAR, MHEALTH, SisFall	
Cao et al., 2024 [133]				DL	CNN, GRU	UCI HAR, HAR70PLUS, HABRD	Dynamic
Kepski et al., 2018 [134]	A, G, M, C		250 Hz	ML	SVM, KNN	URFD	
Kwolek et al., 2014 [25]	A, C	Lower back, Ext	256 Hz	ML	SVM	URFD	Dynamic
Li et al., 2018 [73]	A, C	Waist, Ext	50 Hz	ML, Threshold	SVM, Threshold	Custom	
Sucerquia et al., 2018 [135]	A, C	Waist	±16 g, 200 Hz	Threshold	Threshold	SisFall	

**Sensor type abbreviation**—A: Accelerometer; A + R: Antennas and Receiver; AS: Acoustic sensor; AV: Angular Velocity; C: Camera; ECG: Electrocardiogram; EMG: Electromyography; G: Gyroscope; IR: Infrared sensor; M: Magnetometer; Mag: magnetic sensor; O: Orientation sensor; P: Pressure Sensors; R: Radar; T: Thermal sensor; V: Vibration sensor. **Sensor position abbreviation**—Ext: exterior sensor. **Method abbreviation**—DL: Deep Learning; ML: Machine Learning. **Algorithms abbreviation**—ANN: Artificial Neural Network; AEC: auto-encoder; CNN: Convolutional Neural Network; DT: Decision Tree; GRU: Gait Recurrent Unit; HML: Hybrid Machine Learning; HMM: Hidden Markov Model; KNN: k-Nearest Neighbors; LR: Linear regression; LSTM: Long Short-Term Memory; MLP: Multi-layer Perceptron; NB: Naive Bayes; RF: Random Forest; SVM: Support Vector Machines. **Input data type abbreviation**—Dyn: Dynamic; Stat: Static.

**Table 3:** Performance parameters present in each of the 80 included studies. Data are classified by sensor category: wearable sensors, non-wearable sensors, and hybrid solutions.

Authors	Accuracy	Specificity	Sensitivity	Precision	F1-Score	Training Time	Testing Time
**Wearable sensors**							
Agrawal et al., 2023 [77]	X	X	X				
Al-Hassani et al., 2023 [43]	X	X		X	X		
Ankalaki et al., 2024 [78]	X					X	
Bourke et al., 2007 [79]		X					
Bourke et al., 2008 [80]	X	X	X				
Butt et al., 2021 [81]	X						
Chandramouli et al., 2024 [82]	X						
Chelli et al., 2019 [83]	X			X			
Chen et al., 2018 [84]		X	X				
Gibson et al., 2016 [47]	X	X	X	X	X		
Gulati et al., 2021 [49]	X	X		X	X	X	X
He et al., 2016 [34]		X	X				
He et al., 2017 [85]	X	X	X				
Jahanjoo et al., 2020 [15]		X	X				
Jantaraprim et al., 2012 [86]		X	X				
Kerdegari et al., 2015 [87]	X	X	X				
Khojasteh et al., 2018 [88]	X	X	X	X			
Kraft et al., 2020 [89]	X	X		X	X		
Liaqat et al., 2021 [90]	X	X		X	X		
Martins et al., 2022 [91]	X	X	X		X	X	X
Mauldin et al., 2018 [41]	X		X	X			
Medrano et al., 2017 [92]		X	X				
Miah et al., 2024 [93]	X		X	X	X		
Nyan et al., 2008 [94]		X	X				
Özdemir et al., 2014 [76]	X	X	X			X	X
Özdemir et al., 2016 [95]	X		X				
Pan et al., 2021 [96]	X	X	X				
Putra et al., 2018 [97]			X	X	X		
Rashidpour et al., 2016 [98]		X	X				
Ren et al., 2016 [99]	X	X	X				
Rescio et al., 2018 [100]		X	X				
Sabatini et al., 2016 [101]		X	X				
Santos et al., 2019 [38]	X	X	X	X			
Sarabia-Jácome et al., 2020 [102]	X	X	X				
Shahzad et al., 2018 [103]	X	X	X				
Suriani et al., 2018 [104]	X						
Torti et al., 2019 [42]	X	X	X				
Tunca et al., 2019 [105]	X						
Wu et al., 2018 [64]		X	X				
Xi et al., 2017 [106]		X	X				X
Yacchirema et al., 2018 [35]	X	X	X			X	X
Yoo et al., 2018 [107]	X	X	X				
Yuwono et al., 2012 [108]			X				
**Not wearable sensors**							
Adnan et al., 2018 [109]	X		X	X	X		
Alam et al., 2023 [110]	X	X	X	X	X		
Berlin et al., 2022 [48]	X	X	X	X	X		
de Miguel et al., 2017 [111]	X	X	X	X			
Ding et al., 2023 [20]	X	X		X	X	X	X
Droghini et al., 2017 [46]					X		
Droghini et al., 2017 [112]					X		
Fan et al., 2017 [113]	X						
Guerra et al., 2020 [114]	X	X	X	X			
Guerra et al., 2022 [115]		X	X	X			
Guerra et al., 2023 [17]	X						
Helen Victoria et al., 2021 [116]	X						
Hu et al., 2014 [117]		X	X				
Huu et al., 2022 [118]	X					X	X
Karayaneva et al., 2023 [119]	X						
Li et al., 2012 [120]	X	X	X				
Li et al., 2018 [121]	X	X	X				
Li et al., 2020 [122]	X						
Li et al., 2021 [123]	X						
Li et al., 2023 [124]	X	X		X	X		
Liu et al., 2019 [28]		X	X				
Martelli et al., 2014 [125]	X	X	X				
Min et al., 2018 [126]	X		X	X			
Min et al., 2018 [127]	X						
Natarajan et al., 2025 [128]		X	X	X			
Qiao et al., 2022 [129]	X	X	X	X			
Singh et al., 2019 [22]	X	X		X	X	X	X
Spasova et al., 2016 [130]	X	X	X				
Wang et al., 2020 [19]	X	X		X			
Zahan et al., 2021 [131]	X	X	X		X	X	
**Hybrid solution**							
Alabdulkree et al., 2023 [132]	X						
Benhaili et al., 2025 [45]	X						
Cao et al., 2024 [133]	X				X		
Kepski et al., 2018 [134]	X				X		
Kwolek et al., 2014 [25]	X	X	X	X			
Li et al., 2018 [73]	X						
Sucerquia et al., 2018 [135]	X	X	X				

All studies marked with the symbol X reported at least one value for the performance parameter considered. In many cases, the number of data reported is greater than 1, given the comparisons made between several methods and algorithms. Details of the data are available in the Supplementary File.

**Table 4:** Studies reporting one or more fall detection methods with a performance of at least one parameter equal to 100%. Data are classified by sensor category: wearable sensors, non-wearable sensors, and hybrid solutions.

Authors	Accuracy	Specificity	Sensitivity	Precision	F1-Score	Datasets
**Wearable sensors**						
Bourke et al., 2007 [79]		100% (1)				Custom
Bourke et al., 2008 [80]	100% (1)	100% (1)	100% (1)			Custom
Chelli et al., 2019 [83]				100% (1)		Custom
Jahanjoo et al., 2020 [15]		100% (4)	100% (3)			tfall, MobiFall
Khojasteh et al., 2018 [88]			100% (1)			UMAFall
Mauldin et al., 2018 [41]			100% (2)			Smartwatch, Notch, Farseeing
Nyan et al., 2008 [94]		100% (1)				Custom
Özdemir et al., 2014 [76]			100% (2)			Custom
Pan et al., 2021 [96]	100% (1)	100% (1)	100% (3)			Custom
Putra et al., 2018 [97]			100% (4)			Cogent, Sisfall
Rashidpour et al., 2016 [98]		100% (1)	100% (1)			MobiFall
Sabatini et al., 2016 [101]		100% (1)				Custom
Santos et al., 2019 [38]		100% (3)		100% (3)		URFD, Notch, Smartwatch
Yacchirema et al., 2018 [35]		100% (2)	100% (2)			SisFall
Yoo et al., 2018 [107]	100% (4)	100% (5)	100% (4)			Custom
**Not wearable sensors**						
Alam et al., 2023 [110]		100% (1)		100% (1)		Custom
Berlin et al., 2022 [48]	100% (1)	100% (2)	100% (1)	100% (2)	100% (1)	URFD, FDD
Droghini et al., 2017 [112]					100% (1)	Custom
Li et al., 2012 [120]			100% (1)			Custom
Li et al., 2018 [121]			100% (1)			Custom
Natarajan et al., 2025 [128]			100% (1)			Custom
Singh et al., 2019 [22]		100% (3)				Custom
Wang et al., 2020 [19]	100% (2)	100% (1)				Custom
**Hybrid solution**						
Kepski et al., 2018 [134]	100% (1)				100% (1)	URFD
Kwolek et al., 2014 [25]			100% (2)			URFD

The number in brackets indicates the number of methods/algorithms for each parameter in each study.

## Data Availability

The original contributions presented in this study are included in the article. Further inquiries can be directed to the corresponding author.

## Supplementary Materials

The following supporting information can be downloaded at: https://www.mdpi.com/article/10.3390/s25216540/s1, Table S1: Performance data of all methods presented in each included study used in the meta-analysis.

## Abbreviations

The following abbreviations are used in this manuscript:

A	Accelerometer
A + R	Antennas and Receiver
AAL	Ambient Assisted Living
Acc	Linear dichroism
AEC	auto-encoder
ANN	Artificial Neural Network
AS	Acoustic sensor
AV	Angular Velocity
C	Camera
CNN	Convolutional Neural Network
DL	Deep Learning
DT	Decision Tree
Dyn	Dynamic
ECG	Electrocardiogram
EMG	Electromyography
Ext	exterior sensor.
F1	F1-score
G	Gyroscope
GRU	Gait Recurrent Unit
HML	Hybrid Machine Learning
HMM	Hidden Markov Model
IR	Infrared sensor
KNN	k-Nearest Neighbor
LR	Linear regression
LSTM	Long Short-Term Memory
M	Magnetometer
Mag	magnetic sensor
ML	Machine Learning
MLP	Multi-layer Perceptron
NB	Naive Bayes
O	Orientation sensor
P	Pressure Sensor
Prec	Precision
PRISMA	Preferred Reporting Items for Systematic reviews and Meta-Analyses
R	Radar
RF	Random Forest
Sens	Sensitivity
Spec	Specificity
Stat	Static
SVM	Support Vector Machines
T	Thermal sensor
V	Vibration sensor
